# Supplementary material for: COMBI-r: A Prospective, Non-Interventional Study of Dabrafenib Plus Trametinib in Unselected Patients with Unresectable or Metastatic BRAF V600-Mutant Melanoma
Source: Cancers (Basel). 2023 Sep 6;15(18):4436. doi: 10.3390/cancers15184436 (PMC10526829; doi:10.3390/cancers15184436)
Supplement: Supplementary file 1 [file cancers-15-04436-s001.zip › cancers-2530573-supplementary.pdf]

Supplementary materials

Supplement to: Carola Berking, Elisabeth Livingstone, Dirk Debus, *et al*. **COMBI-r: a Prospective, Non-Interventional Study of Dabrafenib Plus Trametinib in Unselected Patients with Unresectable or Metastatic BRAF V600-mutant Melanoma**

**Contents**


| | |
|---|---|
| List of all investigators | Page 2 |
| | |
| Figure S1. Patient and trial design flow chart | Page 4 |
| Figure S2. Subgroup analyses of progression-free and overall survival by sex | Page 5 |
| Figure S3. Subgroup analyses of progression-free and overall survival by level increased LDH | Page 6 |
| Figure S4. Subgroup analyses of progression-free and overall survival by CNS metastases | Page 7 |
| Figure S5. Subgroup analyses of progression-free and overall survival by use of concomitant corticosteroids | Page 8 |
| Figure S6. Time until end of therapy (per therapy line) | Page 9 |
| Figure S7. Progression-free and overall survival by disease control ± 6 months after treatment start | Page 10 |
| | |
| Table S1. Classification of prior therapies (per therapy line, multiple entries possible) - Analysis Population | Page 11 |
| Table S2. Concomitant non-drug and drug therapy for melanoma | Page 12 |
| Table S3. Patterns of Best Overall Response per line of therapy – Analysis Population | Page 13 |
| Table S4. Criteria to Assess Response to Therapy radiologically and/or clinically | Page 13 |
| Table S5. Disease Control Rate over Time – Analysis Population | Page 14 |
| Table S6. PFS and OS by LDH, affected organ systems, CNS metastases | Page 14 |
| Table S7. Concomitant intake of corticosteroids (in patients with CNS metastases) | Page 15 |
| Table S8. Duration of therapy by investigator-assessed tumour dynamics (per line of therapy and clinical parameters) | Page 15 |
| | |
| Protocol | Page 16 |

**COMBI-r – List of Additional Investigators**

In addition to the authors, the following investigators (listed in alphabetical order) participated in the study and recruited at least one patient:

| Principle Investigator at site | Institution | Ort |
|---|---|---|
| Dr. med. Andreas Arnold | Dermatology of Dermatology, University Medicine Greifswald | Greifswald |
| Prof. Dr. med. Chalid Assaf | Department of Dermatology and Venerology, HELIOS Klinikum Krefeld | Krefeld |
| Prof. Dr. med. Edgar Dippel | Department of Dermatology, Ludwigshafen Medical Center | Ludwigshafen |
| Dr. med. Pia Dücker | Department of Dermatology, Hospital Dortmund | Dortmund |
| Prof. Dr. med. Steffen Emmert | Department of Dermatology and Venereology, University Medical Center Rostock | Rostock |
| Dr. med. Michael Erdmann | Department of Dermatology, Uniklinikum Erlangen | Erlangen |
| Prof. Dr. med. Klaus Fenchel | Onkologische Praxisklinik Saalfeld | Saalfeld |
| Dr. med. Michael Fluck | Department of Oncology Hornheide, Fachklinik Hornheide | Münster |
| Prof. Dr. med. Thilo Gambichler | Department of Dermatology, Ruhr-University Bochum | Bochum |
| Prof. Dr. med. Christoffer Gebhardt | Department of Dermatology and Venereology, University Medical Center Hamburg-Eppendorf | Hamburg |
| Dr. med. Steven Goetze | Department of Dermatology, University Hospital Jena | Jena |
| Thomas Haalck | Department of Dermatology and Venereology, University Medical Center Hamburg-Eppendorf | Hamburg |
| Prof. Dr. med. Sebastian Haferkamp | Department of Dermatology, University Hospital Regensburg | Regensburg |
| Prof. Dr. med. Jessica Hassel | Department of Dermatology and National Center for Tumor Diseases, University Hospital Heidelberg | Heidelberg |
| Prof. Dr. med. Rudolf Herbst | Department of Dermatology, Helios Klinikum Erfurt | Erfurt |
| Dr. med. Norman-Philipp Hoff | Department of Dermatology, Medical Faculty, University Hospital Düsseldorf | Düsseldorf |
| Dr. med. Wiebke Hollburg | Specialist Outpatient Palliative Care Team "PalliativPartner Hamburg GbR" | Hamburg |
| PD Dr. med. Kjell Matthias Kaune | Dermatology, Dermatosurgery and Allergology Clinic, Bremen-Mitte Hospital | Bremen |
| Prof. Dr. med. Claus-Detlev Klemke | Department of Dermatology, Municipal Hospital of Karlsruhe | Karlsruhe |
| Prof. Dr. med. Yon-Dschun Ko | Department of Oncology and Haematology, Center of Integrated Oncology (CIO), Johanniter Hospital Bonn | Bonn |
| Prof. Dr. med. Lutz Kowalzick | Clinic for Dermatology and Allergology, Helios Klinikum Plauen | Plauen |
| Dr. med. Albrecht Kretzschmar | MVZ Mitte am Johannisplatz, Leipzig | Leipzig (Delitzsch) |
| Dr. med. Dirk Mechtel | Department for Dermatology and Allergology, Heinrich-Braun-Klinikum gGmbH Zwickau | Zwickau |
| Prof. Dr. med. Friedegund Meier | Department of Dermatology, University Hospital Dresden, Dresden | Dresden |
| Dr. med. Mark Meinhold | Department of Dermatology, Klinikum Bayreuth | Bayreuth |

| | | |
|---|---|---|
| PD Dr. med. Frank Meiß | Department of Dermatology and Venereology, University Hospital Freiburg | Freiburg |
| Dr. med. Annett Milling | Department of Dermatology, Frankfurt (Oder) | Frankfurt Oder |
| Dr. med. Rose K.C. Moritz | Department for Dermatology, University Hospital Halle | Halle |
| Dr. med. Stephanie Alissa Müller | Department of Dermatology, Klinikum Darmstadt | Darmstadt |
| Dr. med. Gaston Schley | Department of Dermatology, Helios Klinikum Schwerin | Schwerin |
| PD. Dr. med. Jan Schröder | Clinic for Haematology, Oncology and palliative Medicine, Evangelisches Krankenhaus Mülheim/Ruhr | Muelheim |
| Sabine Sell | SRH Waldklinikum Gera | Gera |
| PD Dr. med. Anca Sindrilaru | Department of Dermatology, University Hospital Ulm | Ulm |
| Prof. Dr. med. Rudolf Stadler | Department of Dermatology, Venereology, Allergology, and Phlebology, Johannes Wesling University Hospital Minden | Minden |
| Prof. Dr. med. Patrick Terheyden | Department of Dermatology, Allergology, and Venereology, University of Lübeck | Lübeck |
| Dr. med. Jörg Thomalla | Haematology and Oncology Center Koblenz | Koblenz |
| Dr. med. Christian Tigges | Clinic for Dermatology, Venerology and Allergology, Helios St. Elisabeth Klinik Oberhausen | Oberhausen |
| Prof. Dr. Michael Tronnier | Department of Dermatology, Venereology and Allergology, Hildesheim Clinic | Hildesheim |
| Prof. Dr. Thomas Tüting | Department of Dermatology, University Hospital Magdeburg | Magdeburg |
| Prof. Dr. med. Jens Ulrich | Department of Dermatology and Allergy, Skin Cancer Center Quedlinburg | Quedlinburg |
| Prof. Dr. med. Jochen Utikal | Department of Dermatology, Venereology and Allergology, University Medical Center Mannheim | Mannheim |
| Prof. Dr. med. Carsten Weishaupt | Department of Dermatology, University Hospital of Muenster | Münster |
| Prof. Dr. med. Julia Welzel | Department of Dermatology and Allergology, University Hospital Augsburg | Augsburg |
| Prof. Dr. med. Jörg Wenzel | Department of Dermatology and Allergy, University Medical Center, Bonn | Bonn |
| Dr. Fabian Ziller | Skin Cancer Center, DRK Krankenhaus Chemnitz | Chemnitz |
| Prof. Dr. med. Prof. h.c. Christos C. Zouboulis | Department of Dermatology, Venereology, Allergology and Immunology, Dessau Medical Center | Dessau |

**Figure S1.** Patient and Trial Design Flow Chart

**CONSORT Diagram:**

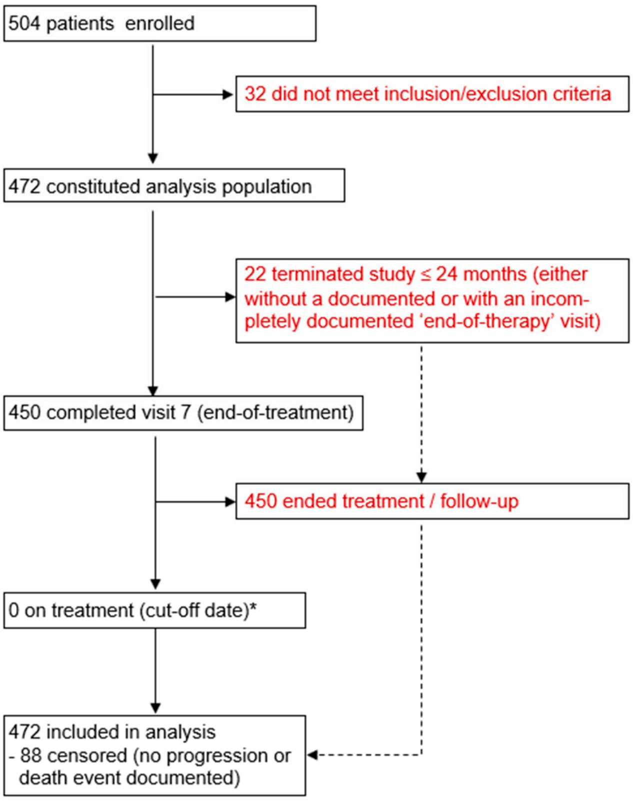


* 16 patients had their last documented visit within 3 months prior to the cut-off date with missing information, if ongoing (i.e., study or treatment)

**Trial design (Overview scheme):**

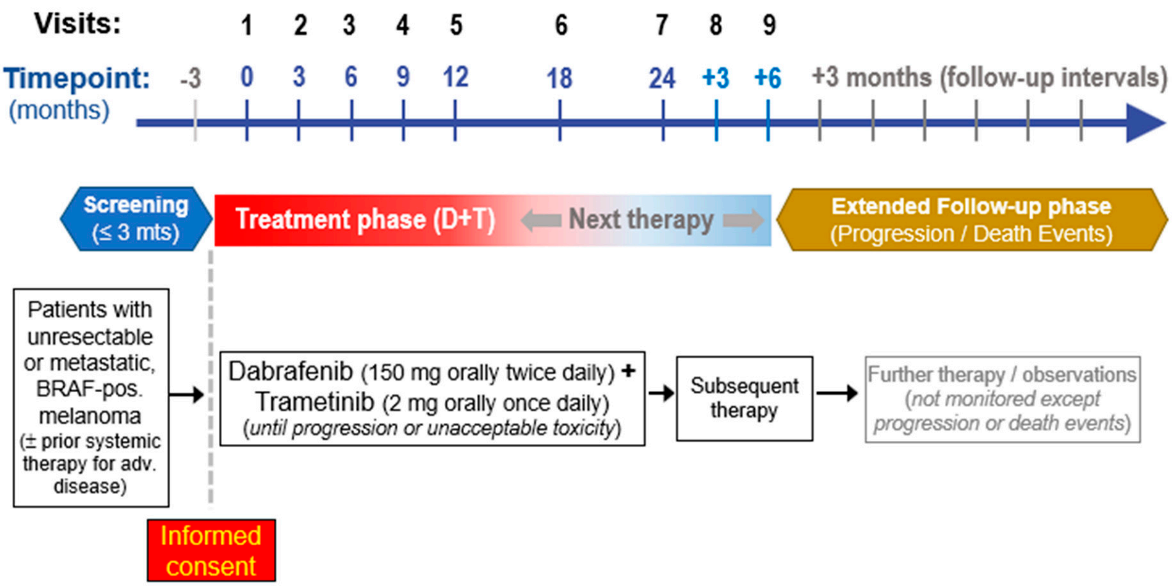

**Figure S2**. **Subgroup analyses of progression-free survival (PFS) and overall survival (OS) by sex**
Cut-off date was 28 July 2021. Median follow-up was 13.5 months. Information on events (yes/no) available for 470 patients for PFS and 464 patients for OS, respectively.

Panel A – Progression-free survival

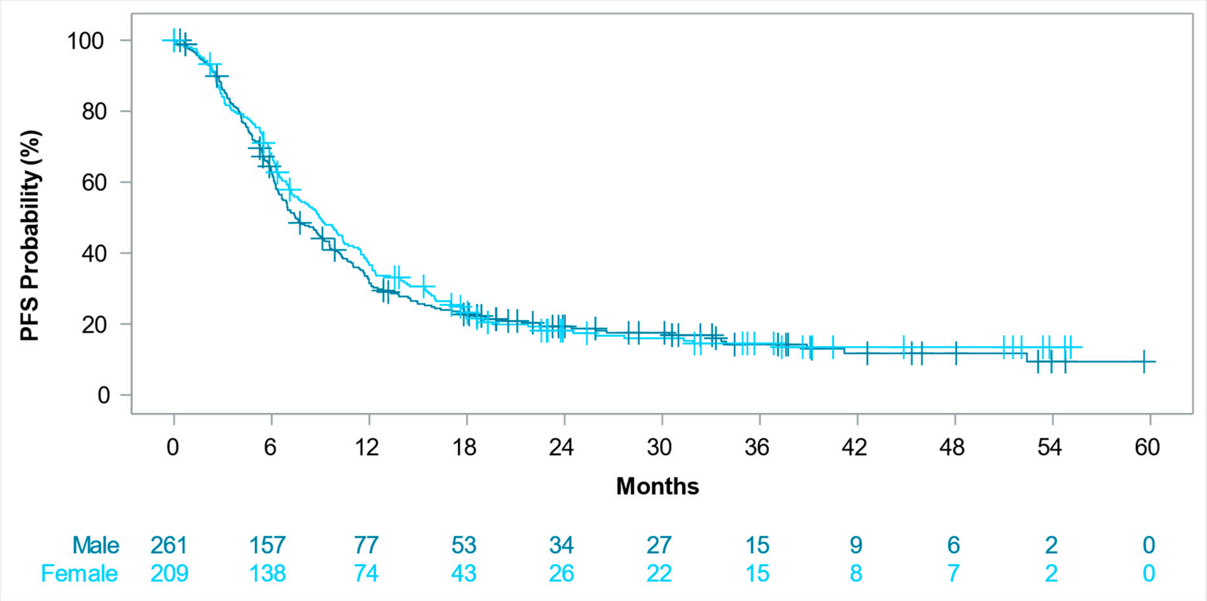


| Gender | Patients, total (n) | PFS Events (n) | % | Median (months) | 95% CL |
|---|---|---|---|---|---|
| Male | 261 | 211 | 80.8 | **7.5** | 6.6-9.2 |
| Female | 209 | 171 | 81.8 | **8.9** | 7.2-10.4 |
| Total | 470 | 382 | 81.3 | **8.3** | 7.1-9.3 |

Panel B – Overall survival

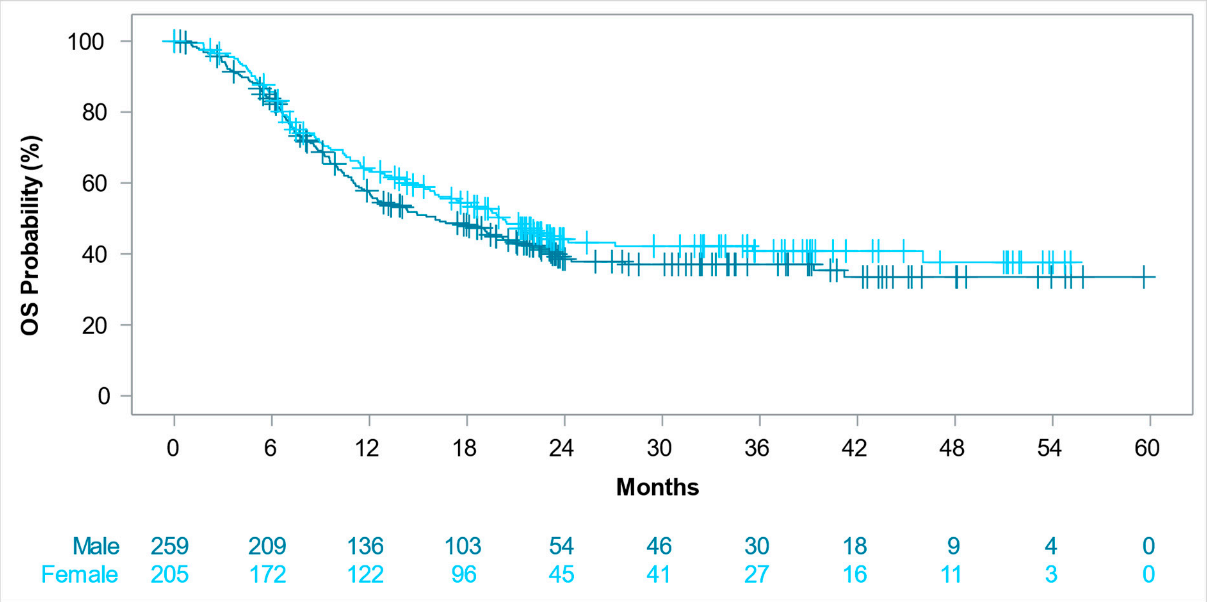


| Gender | Patients, total (n) | OS Events (n) | % | Median (months) | 95% CL |
|---|---|---|---|---|---|
| Male | 259 | 148 | 57.1 | **16.1** | 12.1-20.6 |
| Female | 205 | 109 | 53.2 | **20.2** | 16.0-27.1 |
| Total | 464 | 257 | 55.4 | **18.3** | 14.9-21.3 |

**Figure S3**. **Subgroup analyses of progression-free survival (PFS) and overall survival (OS) by increased LDH**
Cut-off date was 28 July 2021. Median follow-up was 13.5 months. For 77 patients regarding PFS and 86 patients regarding OS, the LDH status (normal vs. elevated) could not be determined, respectively.

Panel A – Progression-free survival

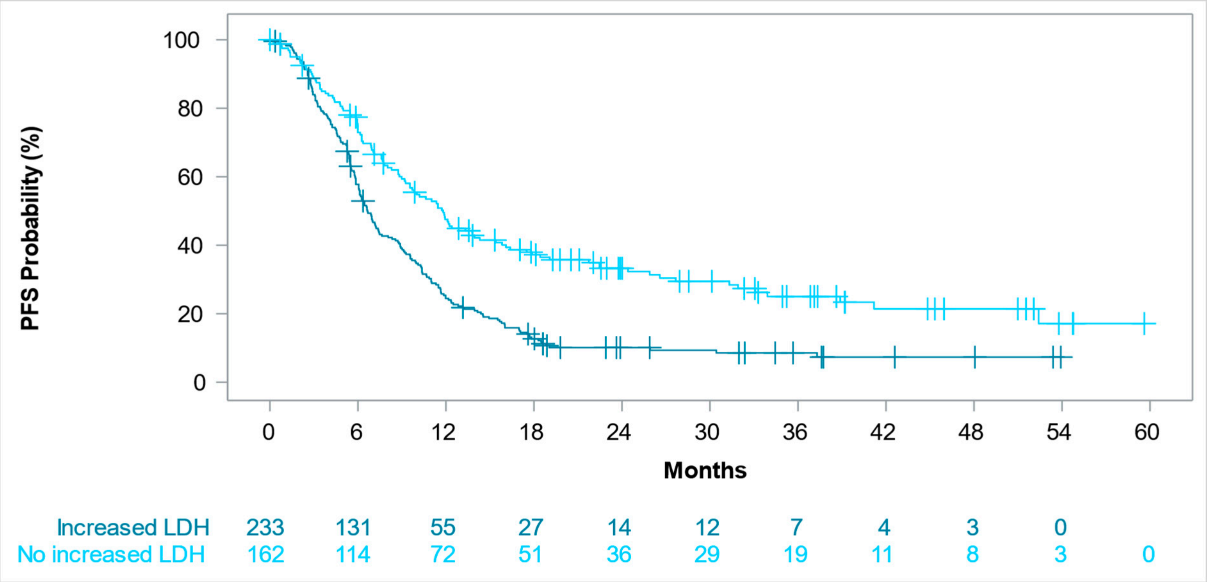


| LDH increased | Patients, total (n) | PFS Events (n) | % | Median (months) | 95% CL |
|---|---|---|---|---|---|
| Yes | 233 | 206 | 88.4 | **6.6** | 6.1-7.4 |
| No | 162 | 113 | 69.8 | **11.8** | 9.2-13.9 |
| Total | 395 | 319 | 80.8 | **8.6** | 7.0-9.5 |

Panel B – Overall survival

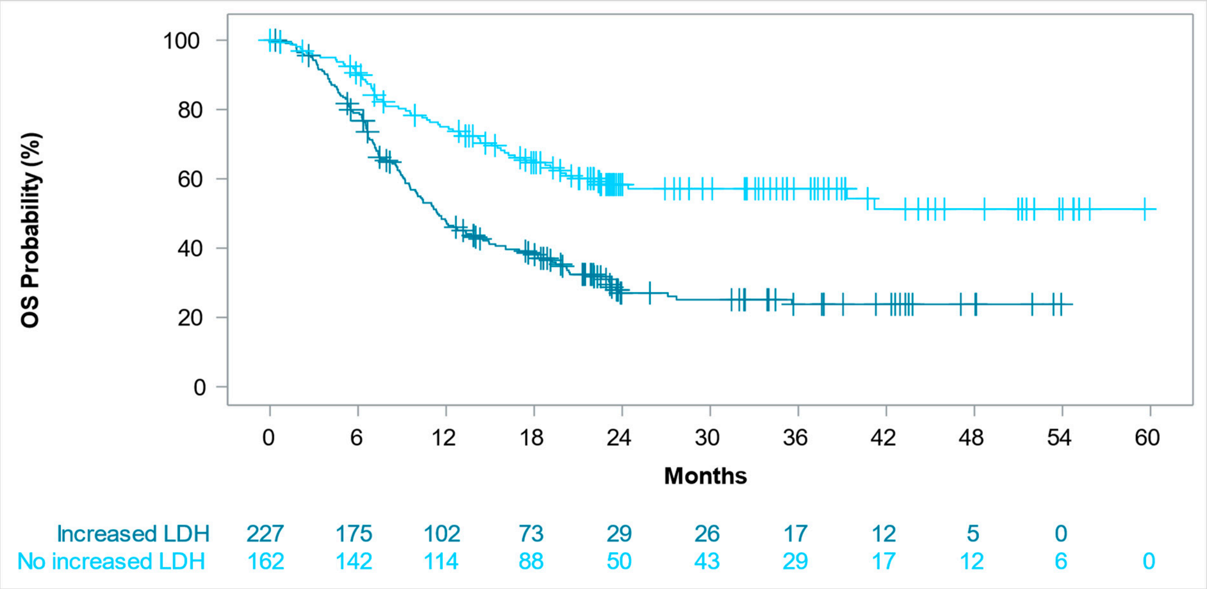


| LDH increased | Patients, total (n) | OS Events (n) | % | Median (months) | 95% CL |
|---|---|---|---|---|---|
| Yes | 227 | 154 | 67.8 | **11.4** | 9.6-14.0 |
| No | 162 | 65 | 40.1 | NR | 22.5-NR |
| Total | 389 | 219 | 56.3 | **17.5** | 14.2-20.4 |

**Figure S4**. **Subgroup analyses of progression-free survival (PFS) and overall survival (OS) by CNS metastases** Cut-off date was 28 July 2021. Median follow-up was 13.5 months. Data for CNS metastases and/or events status were not available for 58 patients regarding PFS and 63 patients regarding OS, respectively.

Panel A – Progression-free survival

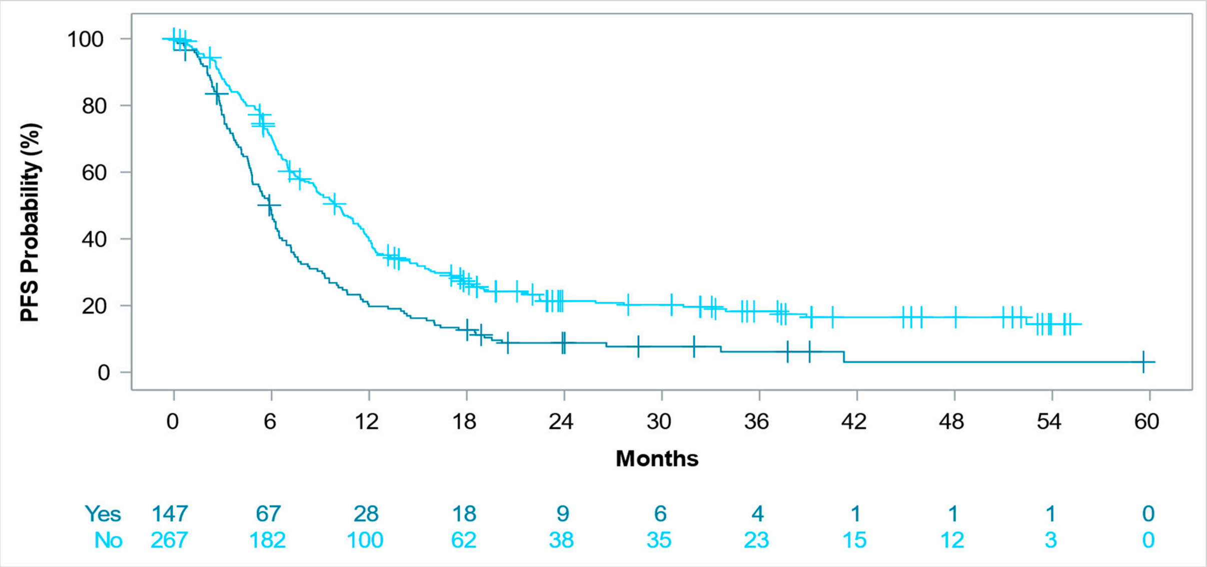


| CNS mets.* | Patients, total (n) | PFS Events (n) | % | Median (months) | 95% CL |
|---|---|---|---|---|---|
| Yes | 147 | 133 | 90.5 | **5.9** | 4.8-6.4 |
| No | 267 | 208 | 77.9 | **10.0** | 8.6-11.3 |
| Total | 414 | 341 | 82.4 | **7.6** | 6.9-9.0 |

Panel B – Overall survival

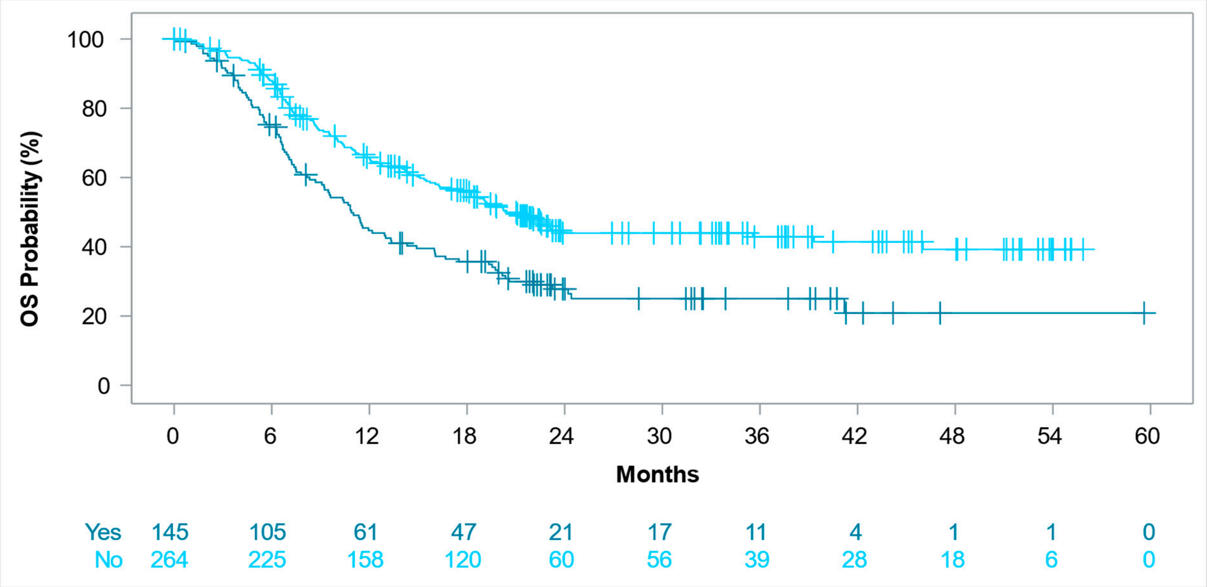


| CNS mets.* | Patients, total (n) | OS Events (n) | % | Median (months) | 95% CL |
|---|---|---|---|---|---|
| Yes | 145 | 101 | 69.7 | **10.9** | 8.7-13.3 |
| No | 264 | 134 | 50.8 | **20.4** | 17.3-35.6 |
| Total | 409 | 235 | 57.5 | **16.7** | 13.7-19.8 |

* CNS metastases at baseline

**Figure S5**. **Subgroup analyses of progression-free and overall survival by use of concomitant corticosteroids**
Cut-off date was 28 July 2021. Median follow-up was 13.5 months. 147 patients had brain metastases, for two of them, no information on survival was available.

Panel A – Progression-free survival

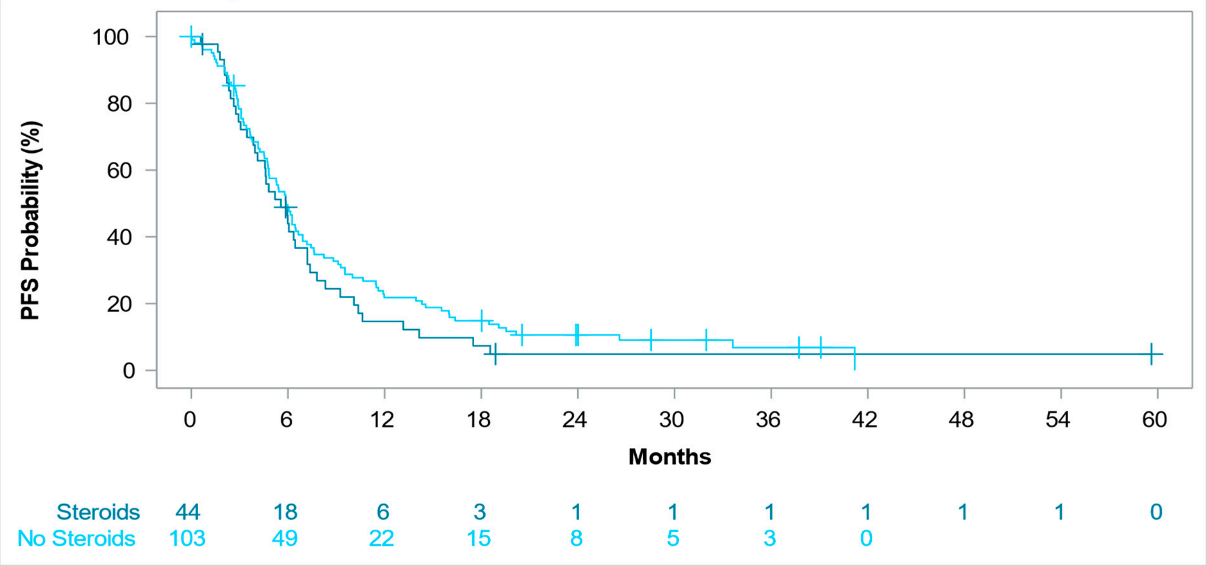


| Corticosteroids req.* | Patients, total (n) | Use Events (n) | % | Median (months) | 95% CL |
|---|---|---|---|---|---|
| Yes | 44 | 40 | 90.9 | **5.6** | 3.9-7.2 |
| No | 103 | 93 | 90.3 | **5.9** | 4.8-6.9 |
| Total | 147 | 133 | 90.5 | **5.9** | 4.8-6.4 |

Panel A – Overall survival

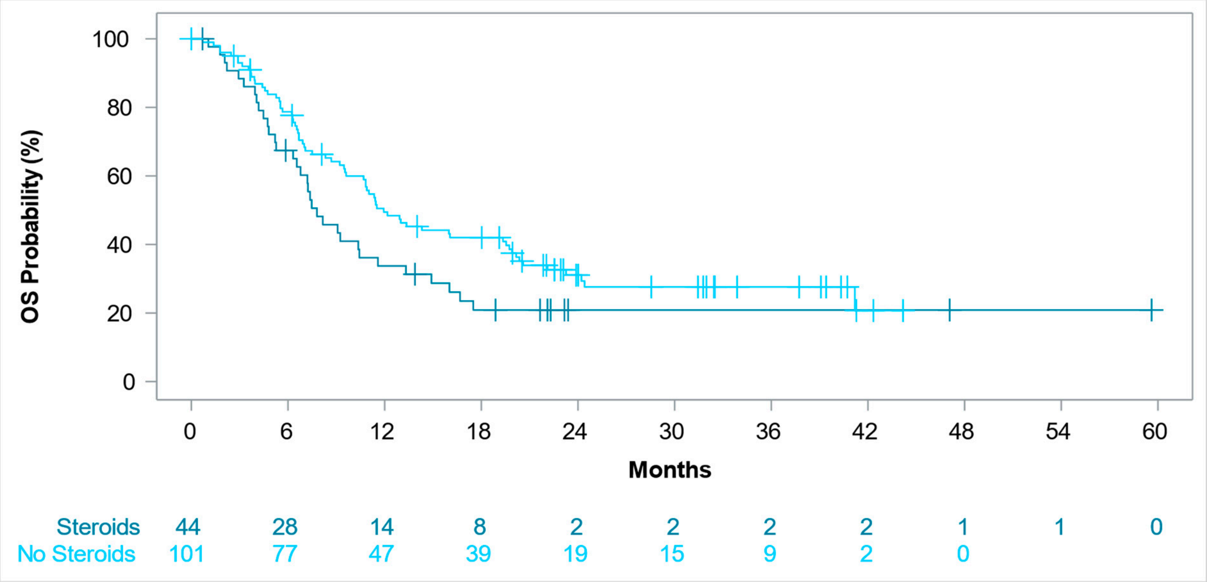


| Corticosteroids req.* | Patients, total (n) | Use Events (n) | % | Median (months) | 95% CL |
|---|---|---|---|---|---|
| Yes | 44 | 33 | 75.0 | **7.8** | 6.3-11.6 |
| No | 101 | 68 | 67.3 | **11.9** | 9.6-19.5 |
| Total | 145 | 101 | 69.7 | **10.9** | 8.7-13.3 |

* Intake of corticosteroids for systemic use at baseline required

**Figure S6. Time until end of therapy (per therapy line)**

Cut-off date was 28 July 2021. Median follow-up was 13.5 months.

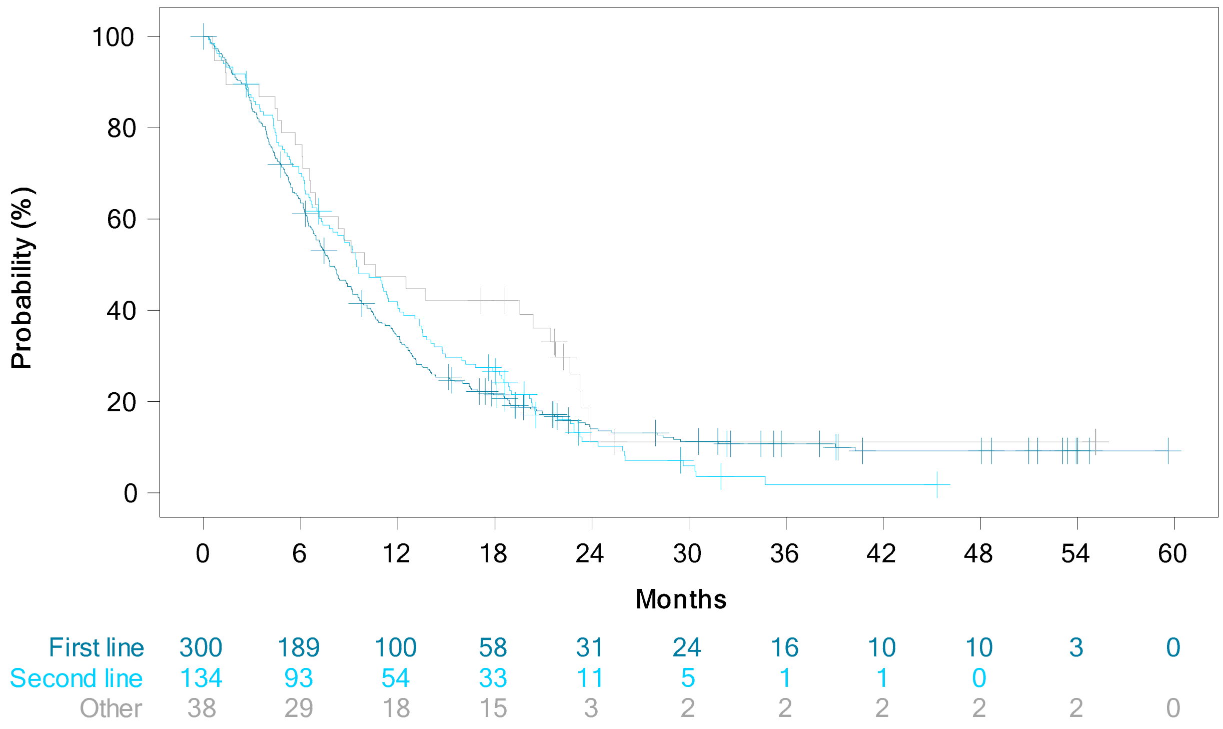


| Line of therapy | Patients, total (n) | Events (n) | % | Median (months) | 95% CI |
|---|---|---|---|---|---|
| First line | 300 | 259 | 86.3 | 7.8 | 6.9-9.2 |
| Second line | 134 | 122 | 91.0 | 9.4 | 7.4-12.0 |
| Other | 38 | 31 | 81.6 | 10.3 | 6.6-21.4 |
| Total | 472 | 412 | 87.3 | 8.7 | 7.5-9.5 |

**Figure S7. Progression-free survival (PFS) and overall survival (OS) by disease control ± 6 months after treatment start**

Cut-off date was 28 July 2021. Median follow-up was 13.5 months. Disease control status ± 6 months after treatment start available in 400 patients regarding PFS and in 395 patients regarding OS.

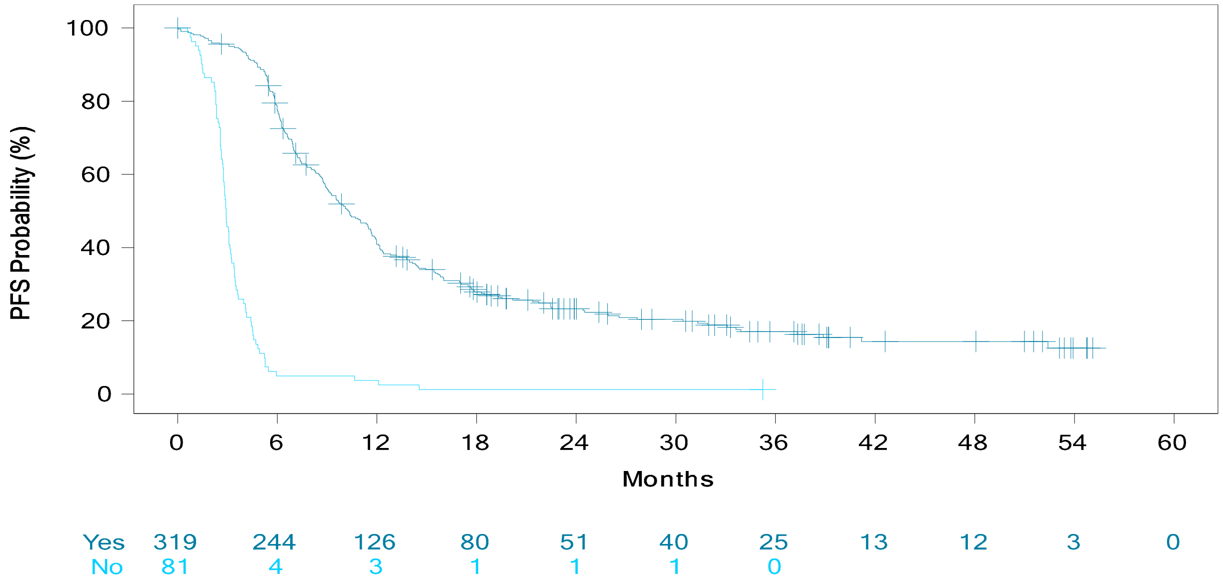


| PFS (by disease control ≥6 months after treatment start) | Patients, total (n) | Events (n) | % | Median (months) | 95% CI |
|---|---|---|---|---|---|
| Yes | 319 | 252 | 79.0 | 10.3 | 9.1-11.6 |
| No | 81 | 80 | 98.8 | 2.9 | 2.8-3.2 |
| Total | 400 | 332 | 83.0 | 8.1 | 6.9-9.1 |

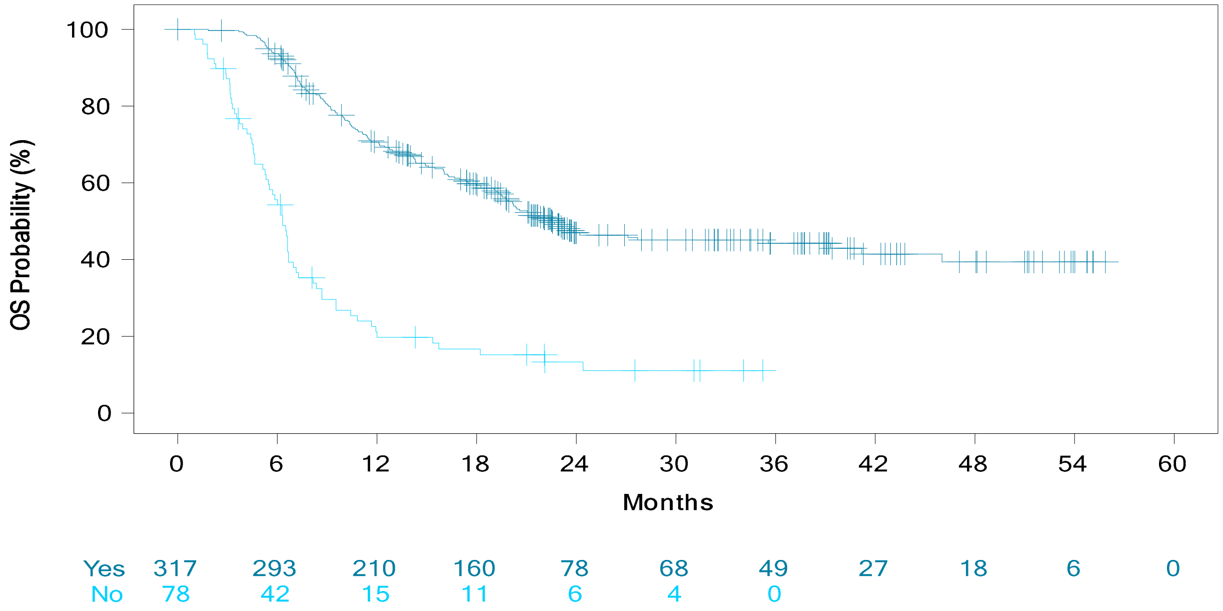


| OS (by disease control ≥6 months after treatment start) | Patients, total (n) | Events (n) | % | Median (months) | 95% CI |
|---|---|---|---|---|---|
| Yes | 317 | 158 | 49.8 | 22.6 | 19.8-39.3 |
| No | 78 | 65 | 83.3 | 6.3 | 5.3-7.0 |
| Total | 395 | 223 | 56.5 | 19.0 | 15.4-21.3 |

**Table S1.** **Classification of prior therapies (by therapy line, multiple entries possible) - Analysis Population**

| | First line n (%) | Second line n (%) | Other lines n (%) | Total n (%) |
|---|---|---|---|---|
| **Total** | 300 (100.0) | 134 (100.0) | 38 (100.0) | 472 (100.0) |
| Prior (drug) therapy* | 69 (23.0) ) | 134 (100.0) | 38 (100.0) | 241 (51.1 ) |
| PD-1/PD-L1 antibodies | 0 (0.0) | 65 (48.5) | 30 (78.9) | 95 (20.1) |
| PD-1 plus CTLA-4 antibodies | 0 (0.0) | 41 (30.6) | 10 (26.3) | 51 (10.8) |
| CTLA4 antibodies | 0 (0.0) | 5 (3.7) | 9 (23.7) | 14 (3.0) |
| BRAF inhibitor (monotherapy) | 0 (0.0) | 8 (6.0) | 15 (39.5) | 23 (4.9) |
| BRAF and MEK inhibitor | 0 (0.0) | 1 (0.7) | 3 (7.9) | 4 (0.8) |
| Other prior (drug) therapies | 69 (23.0) | 51 (38.1) | 21 (55.3) | 141 (29.9) |
| *Antineoplastic agents* | *0 (0.0)* | *8 (6.0)* | *11 (28.9)* | *19 (4.0)* |
| *Adjuvant Interferon therapy*** | *69 (23.0)* | *44 (32.8)* | *17 (44.7)* | *130 (27.5)* |
| *Other Immuno(modulatory) stimulants* | *0 (0.0)* | *6 (4.5)* | *4 (10.5)* | *10 (2.1)* |

* Multiple entries possible

** not counted as line of therapy

**Table S2.** **Concomitant non-drug and drug therapy (by therapy line, multiple entries possible) - Analysis Population**

| | First line<br>n (%) | Second line<br>n (%) | Other lines<br>n (%) | Total<br>n (%) |
|---|---|---|---|---|
| **Total** | 300 (100.0) | 134 (100.0) | 38 (100.0) | 472 (100.0) |
| **Non-drug therapy (any)** | 57 (19.0) | 29 (21.6) | 8 (21.1) | 94 (19.9) |
| *Radiotherapy* | 49 (16.3) | 24 (17.9) | 7 (18.4) | 80 (16.9) |
| *Surgical therapy* | 8 (2.7) | 5 (3.7) | 3 (7.9) | 16 (3.4) |
| *Radiosurgery* | 4 (1.3) | 1 (0.7) | 0 (0.0) | 5 (1.1) |
| *Other therapy* | 1 (0.3) | 0 (0.0) | 1 (2.6) | 2 (0.4) |
| **Pharmacotherapy (any)*** | 99 (33.0) | 45 (33.6) | 14 (36.8) | 158 (33.5) |
| Corticosteroids for systemic use | 36 (12.0) | 21 (15.7) | 2 (5.3) | 59 (12.5) |
| Analgesics | 37 (12.3) | 13 (9.7) | 1 (2.6) | 51 (10.8) |
| Antitumor therapy (antineoplastic agents, immunotherapy)** | 24 (8.0) | 11 (8.2) | 8 (21.1) | 43 (9.1) |
| *Nivolumab* | *15 (5.0)* | *5 (3.7))* | *6 (15.8)* | *26 (5.5)* |
| *Ipilimumab* | *11 (3.7)* | *2 (1.5)* | *4 (10.5)* | *17 (3.6)* |
| *Pembrolizumab* | *5 (1.7)* | *3 (2.2)* | *2 (5.3)* | *10 (2.1)* |
| Drugs for acid-related disorders | 17 (7.5) | 8 (6.0) | 0 (0.0) | 25 (5.3) |
| Drugs for treatment of bone disease | 18 (6.0) | 6 (4.5) | 1 (2.6) | 25 (5.3) |
| Antiepileptics | 10 (3.3) | 7 (5.2) | 0 (0.0) | 17 (3.6) |
| Drugs for functional gastrointestinal disorders | 7 (2.3) | 5 (3.7) | 0 (0.0) | 12 (2.5) |
| Mineral supplements | 4 (1.3) | 4 (3.0) | 2 (5.3) | 10 (2.1) |

* per ATC level (level 2), if drug class was taken by at least 2% in the total population

** partially overlapping with (or immediately following after) treatment period dabrafenib + trametinib (reporting related or due to lacking start/stop dates in case report forms for (‘concomitant’) antitumor therapy)

**Table S3. Patterns of Best Overall Response – Analysis Population**

| Best overall response (EoT) | First line n (%) | Second line n (%) | Other lines n (%) | Total n (%) |
|---|---|---|---|---|
| Complete Remission | 34 (12.0) | 14 (11.0) | 6 (16.2) | 54 (12.1) |
| Partial Remission | 81 (28.5) | 24 (18.9) | 14 (37.8) | 119 (26.6) |
| Stable disease | 62 (21.8) | 35 (27.6) | 11 (29.7) | 108 (24.1) |
| Progressive disease | 64 (22.5) | 35 (27.6) | 3 (8.1) | 102 (22.8) |
| Not assessable | 43 (15.1) | 19 (15.0) | 3 (8.1) | 65 (14.5) |
| Total | 284 (100.0) | 127 (100.0) | 37 (100.0) | 448 (100.0) |

**Table S4. Criteria to Assess Response to Therapy Radiologically and/or Clinically**

| **Response** | **Criteria according to RECIST 1.1** (Eisenhauer *et al.*, 2009) | **Clinical Criteria (might be supported by imaging)** |
|---|---|---|
| Complete response (CR) | Disappearance of all target lesions. Any pathological lymph nodes (whether target or non-target) must have reduction in short axis to <10 mm. | Major improvement of clinical symptoms in line with complete regression of all tumor lesions (palpable/non-palpable, imaging required for latter case). |
| Partial response (PR) | At least a 30% decrease in the sum of diameters of target lesions, taking as reference the baseline sum diameters. | Amelioration of clinical symptoms and/or remission of tumor size (i.e., number and size of lesions). In case, only one of both criteria is fulfilled, the improvement should be judged as clinically relevant. |
| Stable disease (SD) | Neither sufficient shrinkage to qualify for PR nor sufficient increase to qualify for PD, taking as reference the smallest sum diameters while on study. | Neither worsening of condition (in symptoms and/or tumor size) nor amelioration (corresponding to an achievement of a CR or PR). |
| Progressive disease (PD) | At least a 20% increase in the sum of diameters of target lesions, taking as reference the smallest sum on study (this includes the baseline sum if that is the smallest on study). In addition to the relative increase of 20%, the sum must also demonstrate an absolute increase of at least 5 mm. (Note: the appearance of one or more new lesions is also considered progression) | Increase in tumor size (number or size of lesions) and/or worsening of clinical condition/symptoms. |

**Table S5.** **Disease Control Rate over Time – Analysis Population**

| | | Therapy line | | | |
|---|---|---|---|---|---|
| | | **First line n (%)** | **Second line n (%)** | **Other lines n (%)** | **Total n (%)** |
| Controlled disease* **until 6 months** after treatment start | No | 50 (19.5) | 25 (22.3) | 6 (17.6) | 81 (20.1) |
| | Yes | 206 (80.5) | 87 (77.7) | 28 (82.4) | 321 (79.9) |
| | **Total** | 256 (100.0) | 112 (100.0) | 34 (100.0) | 402 (100.0) |
| | *Missing* | *44* | *22* | *4* | *70* |
| Controlled disease* **until 12 months** after treatment start | No | 15 (7.7) | 6 (6.5) | 3 (10.7) | 24 (7.6) |
| | Yes | 180 (92.3) | 86 (93.5) | 25 (89.3) | 291 (92.4) |
| | **Total** | 195 (100.0) | 92 (100.0) | 28 (100.0) | 315 (100.0) |
| | *Missing* | *105* | *42* | *10* | *157* |

* i.e., defined as at least one lasting response (complete or partial response) or disease stabilisation within 6 respectively 12 months of treatment

**Table S6.** **PFS and OS by LDH, affected organ systems, CNS metastases**

| | **Total** | **PFS events** | | **PFS (months)** | | | **OS events** | | **OS (months)** | |
|---|---|---|---|---|---|---|---|---|---|---|
| **At baseline** | **n** | **n** | **%** | **Median** | **95% CI** | **n** | **n** | **%** | **Median** | **95% CI** |
| **Increased LDH** | 233 | 206 | 88.4 | 6.6 | 6.1-7.4 | 227 | 154 | 67.8 | 11.4 | 9.6-14.0 |
| **Normal LDH** | 162 | 113 | 69.8 | 11.8 | 9.2-13.9 | 162 | 65 | 40.1 | NR | 22.5-n/a |
| **Total** | 395 | 319 | 80.8 | 8.6 | 7.0-9.5 | 389 | 219 | 56.3 | 17.5 | 14.2-20.4 |
| **1-2 affected organs** | 224 | 166 | 74.1 | 11.0 | 8.9-12.2 | 222 | 109 | 49.1 | 23.1 | 19.1-41.2 |
| **≥3 affected organs** | 191 | 176 | 92.1 | 6.2 | 5.5-6.6 | 188 | 127 | 67.6 | 10.8 | 9.1-12.5 |
| **Total*** | 415 | 342 | 82.4 | 7.6 | 6.9-9.0 | 410 | 236 | 57.6 | 16.3 | 13.7-19.8 |
| **CNS metastases** | 147 | 133 | 90.5 | 5.9 | 4.8-6.4 | 145 | 101 | 69.7 | 10.9 | 8.7-13.3 |
| **No CNS mets.** | 267 | 208 | 77.9 | 10.0 | 8.6-11.3 | 264 | 134 | 50.8 | 20.4 | 17.3-35.6 |
| **Total*** | 414 | 341 | 82.4 | 7.6 | 6.9-9.0 | 409 | 235 | 57.5 | 16.7 | 13.7-19.8 |

* Patients with clinical stage IV and documented distant metastases only; based on 416 patients (missing data sets due to missing/incorrect dates of progression or death)

**Table S7. Concomitant intake of corticosteroids** (in patients with CNS metastases)

| | Intake of corticosteroids | First line n (%) | Second line n (%) | Other lines n (%) | Total n (%) |
|---|---|---|---|---|---|
| **At baseline** | **Yes** | 30 (33.0) | 13 (27.7) | 1 (11.1) | 44 (29.9) |
| | **No** | 61 (67.0) | 34 (72.3) | 8 (88.9) | 103 (70.1) |
| | **Total** | 91 (100.0) | 47 (100.0) | 9 (100.0) | 147 (100.0) |
| **After 90 days** | **Yes** | 27 (34.2) | 12 (29.3) | 1 (12.5) | 40 (31.3) |
| | **No** | 52 (65.8) | 29 (70.7) | 7 (87.5) | 88 (68.8) |
| | **Total** | 79 (100.0) | 41 (100.0) | 8 (100.0) | 128 (100.0) |

**Table S8. Duration of therapy by investigator-assessed tumour dynamics (per line of therapy and clinical parameters)**

| Variable | Statistic | Fast growth | Intermediate growth | Slow growth | Missing | Total |
|---|---|---|---|---|---|---|
| Number of days with application of dabrafenib | N Valid | 99 | 86 | 32 | 181 | 398 |
| | Median | 191 days | 243 days | 379 days | 190 days | 211 days |
| | Range | 12-902 | 13-1225 | 14-926 | 9-750 | 9-1225 |
| Number of days with application of trametinib | N Valid | 99 | 86 | 32 | 181 | 398 |
| | Median | 190 days | 242 days | 379 days | 188 days | 206 days |
| | Range | 13-902 | 16-1225 | 14-926 | 10-742 | 10-1225 |
| Duration [days] of therapy (DOT) | N Valid | 100 | 87 | 33 | 192 | 412 |
| | Median | 195 | 266 | 393 | 200 | 222 |
| | Range | 13-924 | 87-1225 | 33-926 | 10-1188 | 10-1225 |

**Study protocol**

The study protocol (initial version and amended versions, all in German language) has been previously published as part of the notification to the non-interventional study (NIS no. 6690) to the German regulatory authority and is available at the dedicated website: https://awbdb.bfarm.de/ords/f?p=101:25:::NO::P25_AWB_ID:98396

The final clinical study report (Version 8 Apr 2022, English language version) is available via the German Clinical Trial Register (DRKS): https://drks.de/search/de/trial/DRKS00011387
